# An unusual case of an isolated capitellar fracture of the right elbow in a child: a case report

**DOI:** 10.1186/1752-1947-6-57

**Published:** 2012-02-10

**Authors:** José António Gonçalves Pestana, Ana Patrícia Macedo França, António Pedro Tomás Cunha Freitas, Bruno Tertuliano Jales, Cristina Alves, Fabíola Ferreira, Manuel Correia Ramos, Mário Pereira

**Affiliations:** 1 Department of Orthopedics, Hospital Central do Funchal, Madeira, Portugal

## Abstract

**Introduction:**

Although elbow fractures have a high incidence in the pediatric population, fractures of the capitellum are almost exclusively observed in individuals older than 12 years of age. Due to their rarity in children, reports with large numbers of cases are lacking in the literature and the surgical treatment options are poorly defined.

**Case presentation:**

We present the case of an 11-year-old Portuguese girl with a displaced fracture of the capitellum of the right elbow, a typical Hahn-Steinthal or Type 1 fracture, which was followed for one year. The treatment and outcome of this fracture are described. Our patient underwent an open reduction and internal fixation with two cannulated screws. There were no complications and normal elbow function was recovered.

**Conclusion:**

The authors believe that cannulated screw fixation is a reliable method of treatment for Type 1 capitellar fracture in children because it enables good interfragmentary compression, early mobilization, faster functional elbow recovery and implant removal is rarely necessary.

## Introduction

Isolated capitellar fractures are rare and represent only 1% of all elbow fractures and 6% of distal humeral fractures [1-3]. In children under the age of 12, these fractures are even more unusual and, to the best of our knowledge, such an injury has not been reported in a young child [3-8]. The mostly cartilaginous composition of the capitellum below that age makes it more resistant to stress. Therefore, a fall on the outstretched hand (which is the mechanism of injury in most of these cases) is more likely to produce a supracondylar or lateral condylar fracture. As the capitellum grows and ossifies in older children, it becomes more susceptible to shear injury [5,9]. Fracture of the lateral humeral condyle is usually seen at a younger age group and may be confused with a fracture of the capitellum. A fracture of the capitellum is an osteochondral fracture and is entirely intra-articular. The epicondyle, the growth plate and the metaphysis are not involved, and the posterior part of the lateral condyle remains intact. Differentiation between a fracture of the lateral humeral condyle and a capitellar fracture is important with respect to treatment [7,8].

**Figure 1:**
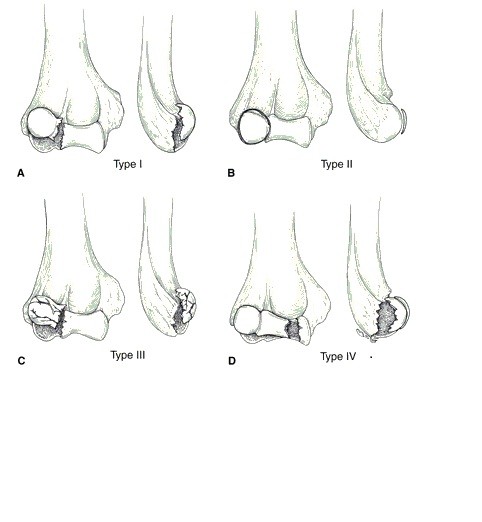
**Bryan and Morrey classification of capitellar fracture**.

The presently accepted classification systems of capitellar fractures are descriptive and not treatment-directed [10]. Bryan and Morrey classified capitellar fractures as Type 1, 2 and 3 [1]. Type 1, often referred to as the Hahn-Steinthal fracture, is a shear fracture in the coronal plane involving most of the capitellum and little or none of the trochlea. Type 2, called the Kocher-Lorenz fracture, involves a variable amount of articular cartilage of the capitellum with minimal attached subchondral bone. Type 3 is a comminuted or compression fracture of the capitellum. McKee and colleagues described a fourth type, a shear fracture of the distal aspect of the humerus in the coronal plane including the capitellum and most of the trochlea (Figure 1) [11]. Ring and colleagues reported another descriptive classification for distal humeral articular fractures and, recently, Dubberley and colleagues proposed a novel classification system [12]. As a result of the rarity of capitellar fractures in children, studies focusing on this injury are insufficient. Furthermore, the administration of treatment for this type of injury remains controversial with regards to young children [5-8,13].

In adults, a variety of treatment methods have been described. As this type of fracture became better recognized in the adult population, treatment options evolved from closed reduction and immobilization, or excision of the capitellar fragment, to open reduction and internal fixation in order to achieve a stable anatomic reduction [2,3,10-17].

We present the case of an 11-year-old patient with a Hahn-Steinthal fracture treated by open reduction and internal fixation with two cannulated screws.

## Case presentation

**Figure 2:**
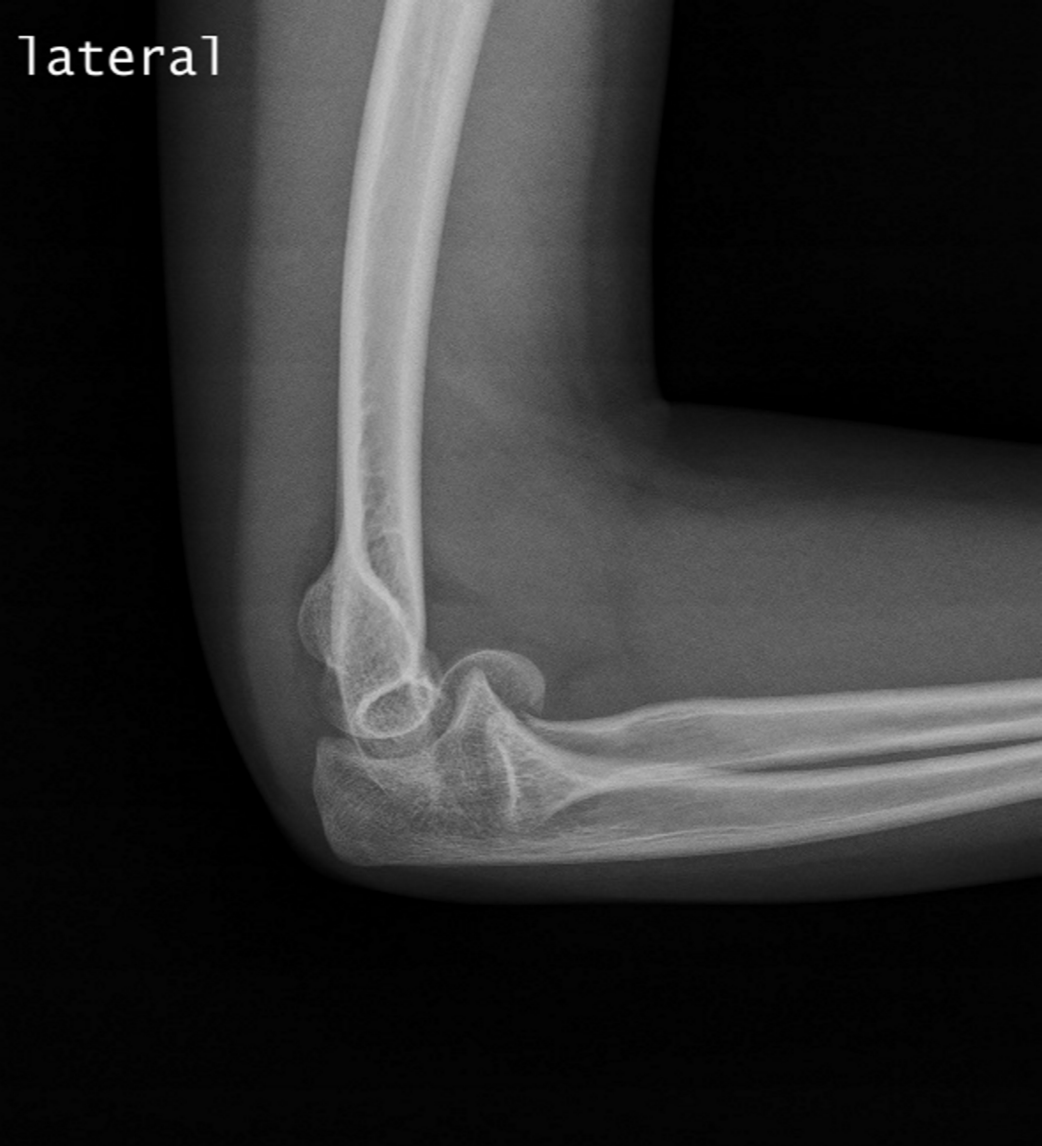
**Right Hahn-Steinthal Type 1 capitellum fracture, preoperative lateral radiograph**.

**Figure 3:**
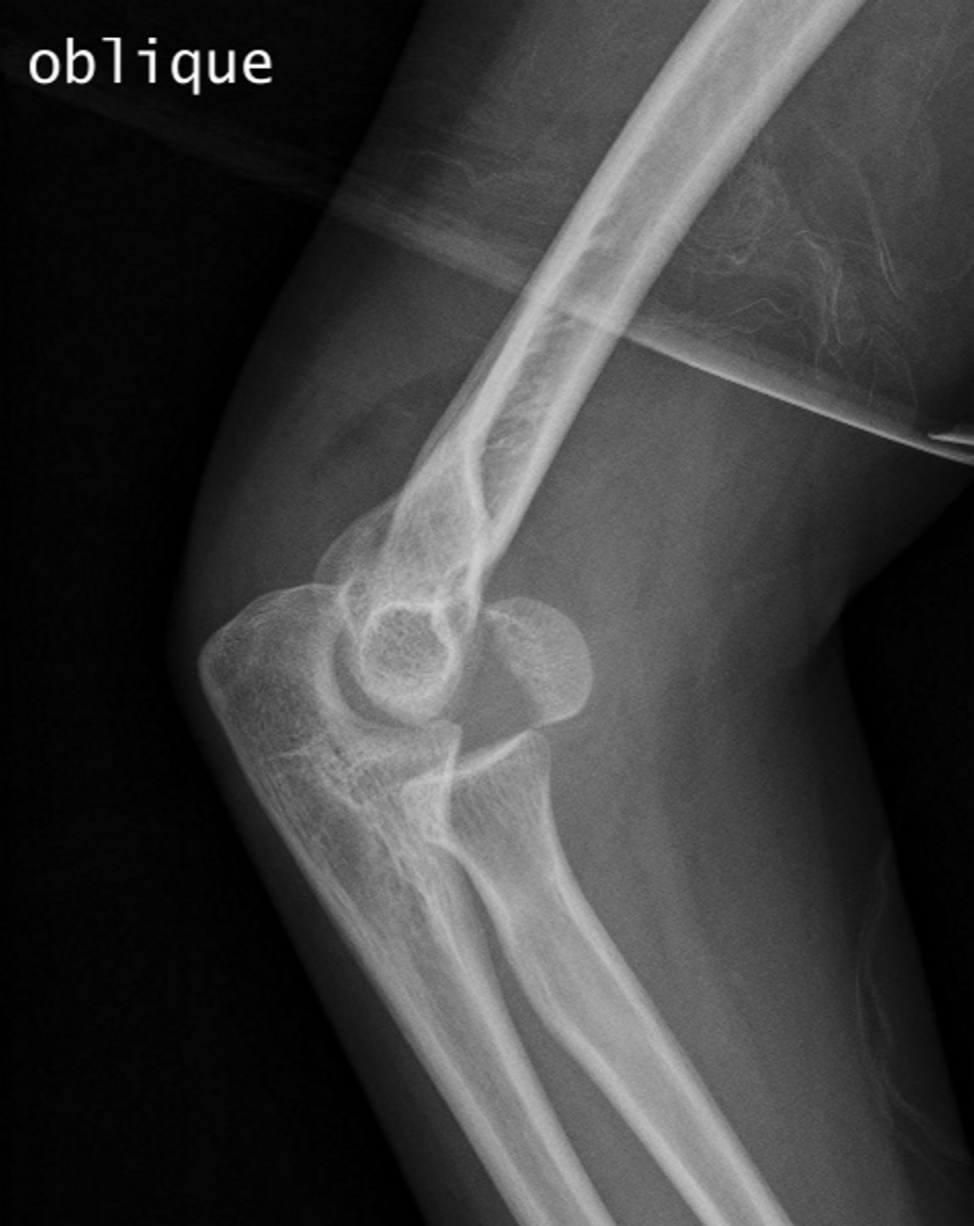
**Right Hahn-Steinthal Type 1 capitellum fracture, preoperative oblique radiograph**.

We describe the case of an 11-year-old right-hand-dominant White Portuguese girl who presented with pain in her right elbow following a fall onto the outstretched hand that occurred while she was riding a bicycle. She was unable to move her elbow actively due to pain. A physical examination revealed mild swelling and tenderness over the lateral aspect of the distal part of her humerus. The child had a restricted range of motion of her elbow, especially with flexion. Joint stability was considered normal and no neurovascular signs were observed. The diagnosis was made on a lateral and an oblique radiograph (Figures 2 and 3), which revealed a displaced fracture of the capitellum of her right elbow, a typical Hahn-Steinthal or Type I fracture. An anteroposterior radiograph of the distal part of her right humerus did not reveal a definite fracture. No other injuries were detected.

**Figure 4:**
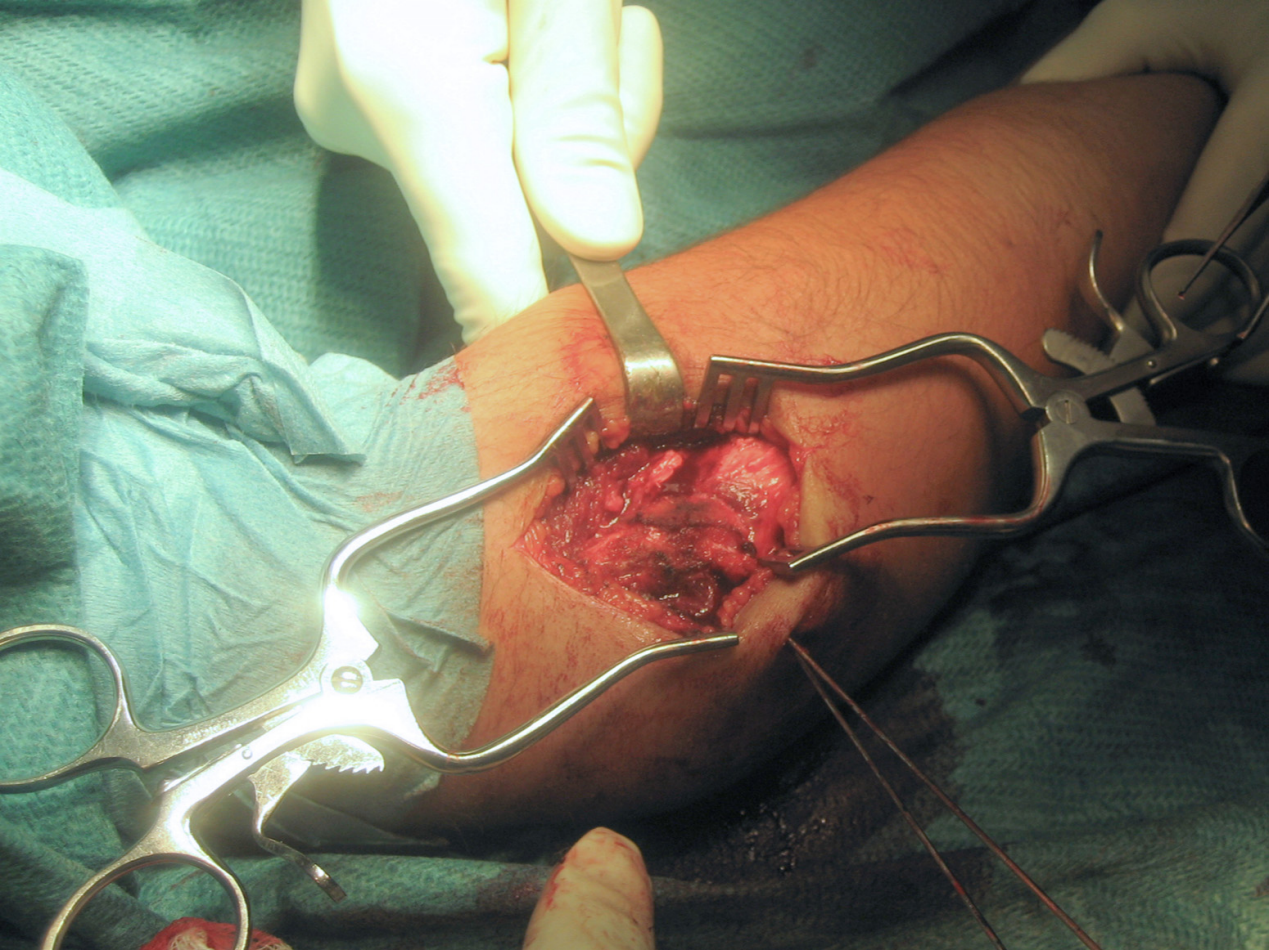
**Posterolateral approach to her right elbow**.

**Figure 5:**
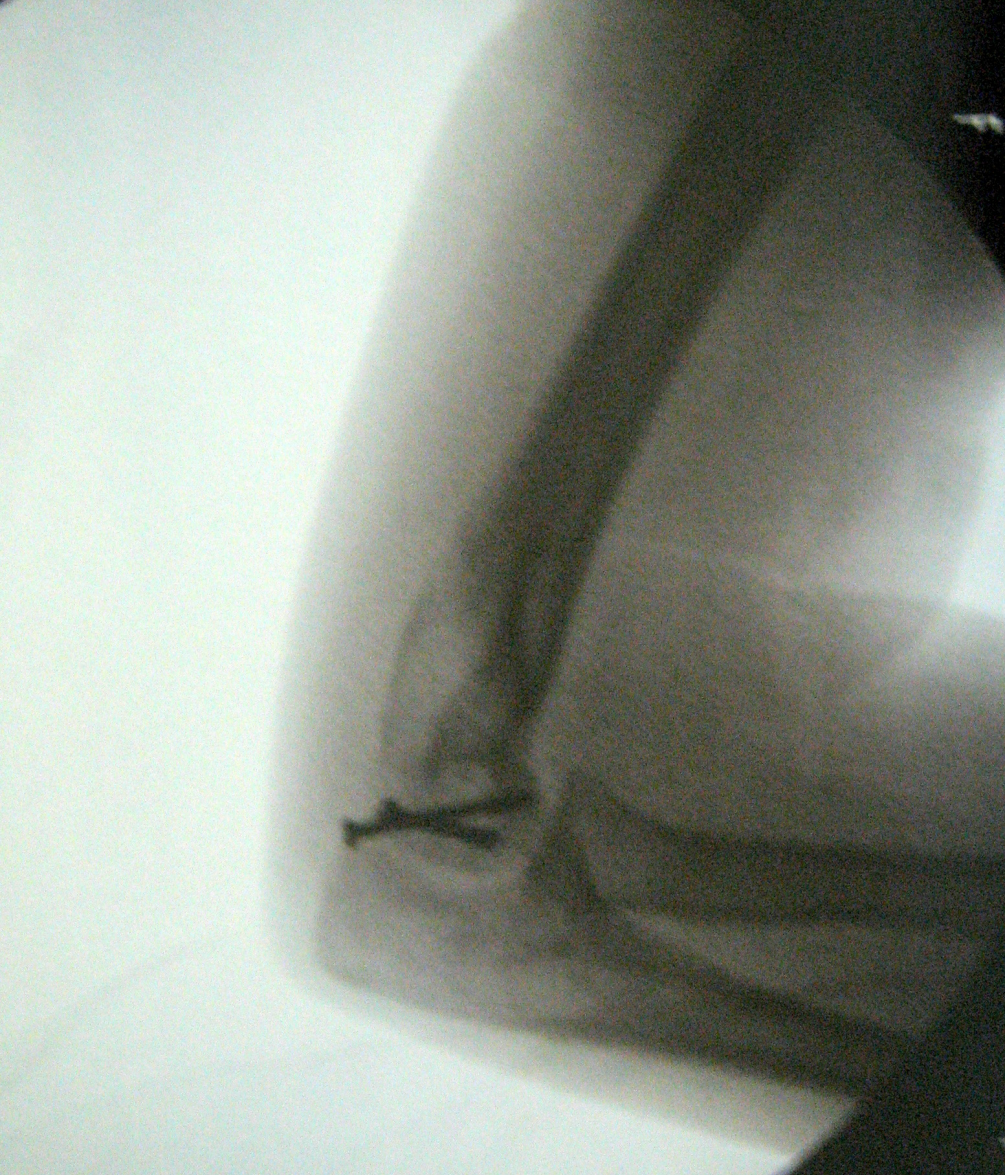
**Intraoperative lateral fluoroscopic image of the cannulated screws**.

Our patient was treated by open reduction and internal fixation. Under general anesthesia, an attempt was made at closed reduction but complete reduction could not be achieved that way. With a pneumatic tourniquet in place, a posterolateral incision was made (Figure 4). Further manipulation of the fragment allowed reduction that was held in the correct position with two temporary Kirschner wires. The final fixation was performed with two 3.0 mm cannulated screws. The screws were inserted in a posteroanterior direction through the posterior surface of the lateral condyle. An intraoperative dynamic examination showed satisfactory stability of the osteosynthesis and anatomic articular congruity. A protrusion of the tip of the screw on the articular surface was carefully avoided (Figure 5). Postoperatively, her elbow was immobilized in a long arm cast at a right angle, with the forearm in a neutral position for three weeks. The postoperative outcome was uneventful, allowing our patient to leave the hospital 48 hours after surgery.

The follow-up period was of one year with regular clinical examination and roentgenographic control. Our patient was asked about pain and daily activities. Her elbow flexion-extension and pronation-supination were evaluated and compared with the contralateral side.

**Figure 6:**
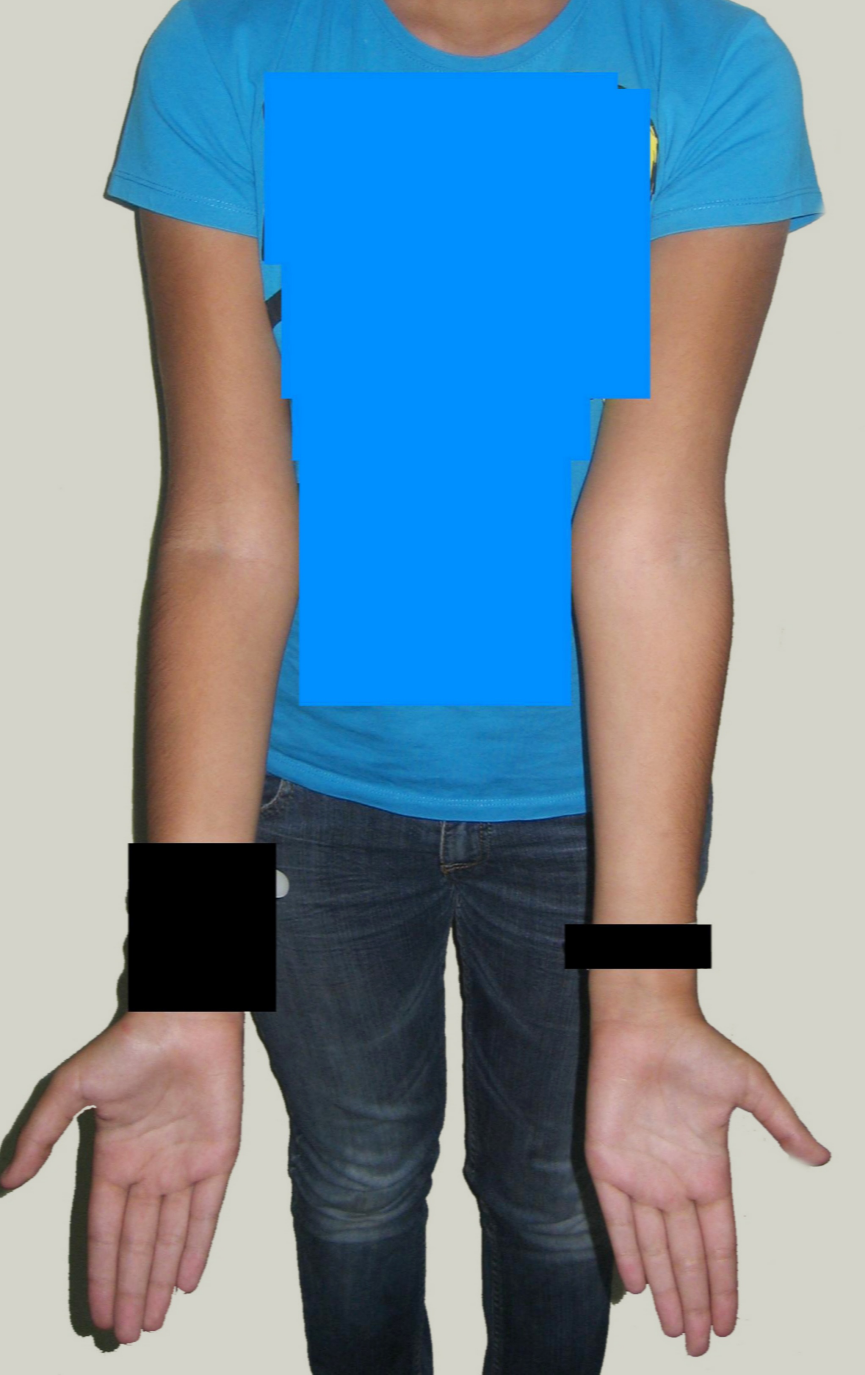
**Good clinical elbow outcome at the four month follow-up evaluation; extension**.

**Figure 7:**
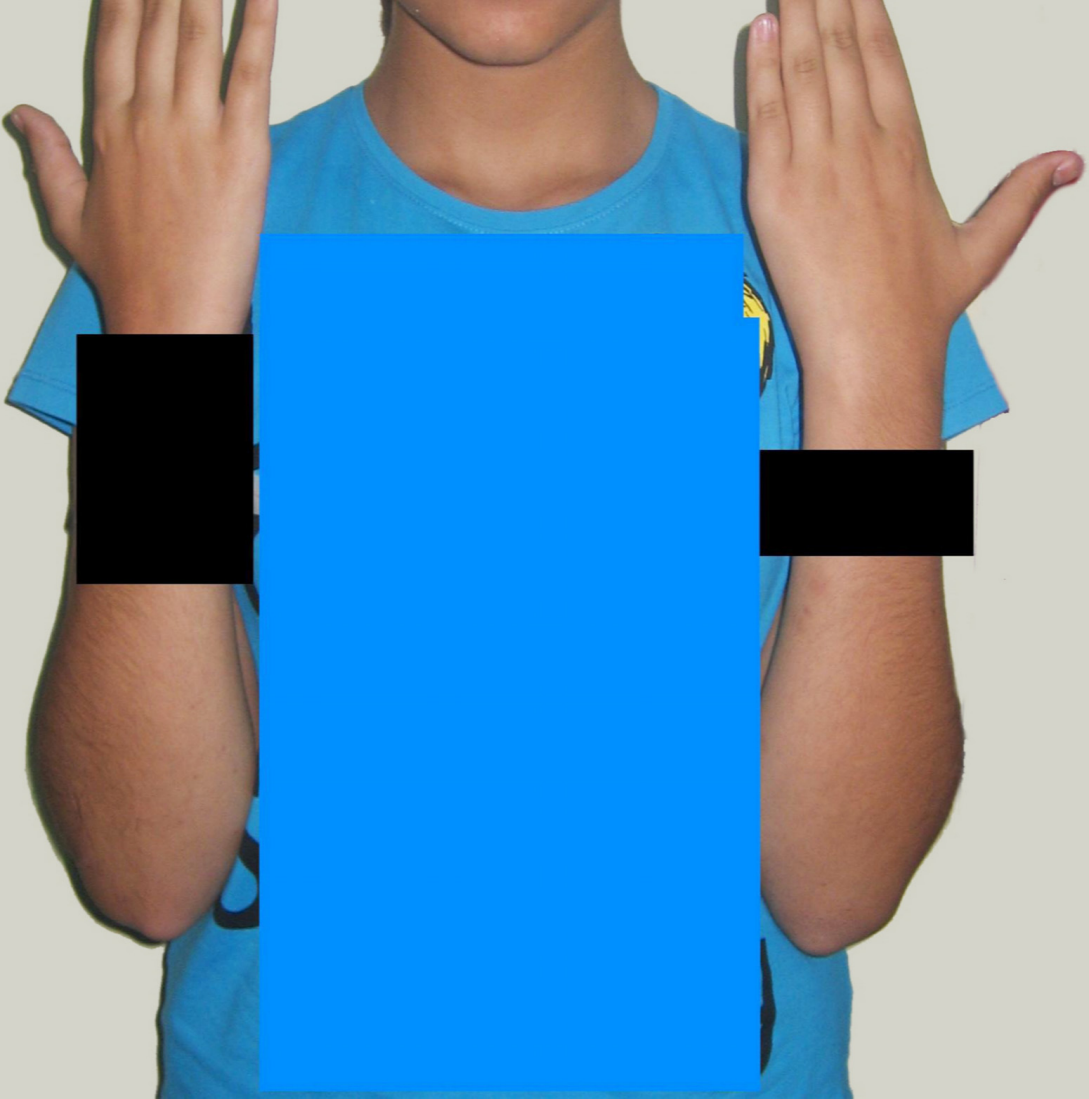
**Good clinical elbow outcome at the four month follow-up evaluation; flexion-supination**.

**Figure 8:**
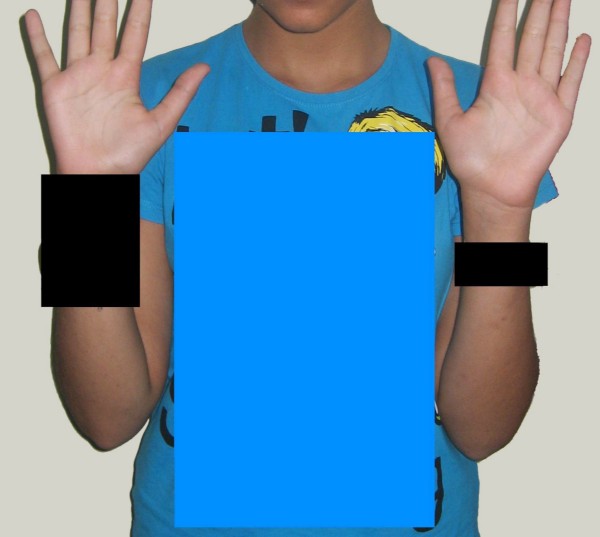
**Good clinical elbow outcome at the four month follow-up evaluation; flexion-pronation**.

**Figure 9:**
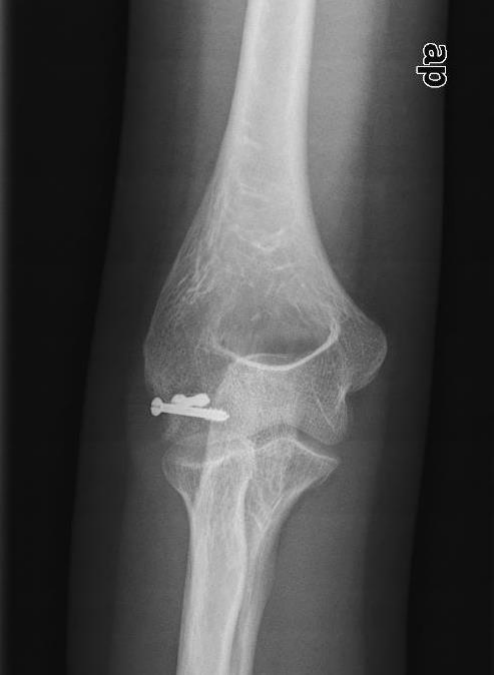
**Anteroposterior radiograph one year after the surgery, showing the fracture consolidation**.

**Figure 10:**
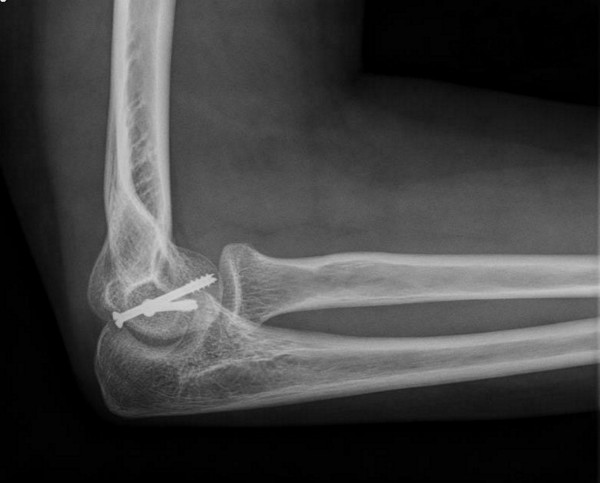
**Lateral radiograph one year after the surgery, showing the fracture consolidation**.

Our patient was able to perform normal elbow movements and normal daily activities without pain, but she mentioned having limited mobility after removal of the plaster cast. This initial period was followed by four months of a progressive mobilization program that was guided by a physiotherapist. On the basis of the radiographic appearance, the fracture presented convincing signs of consolidation after five weeks. At the fourth month of follow-up, the fracture was considered completely healed and our patient had a good range of motion with full flexion, pronation and supination, but lacked 15° of extension in comparison with her contralateral side (Figures 6, 7 and 8). There was no varus or valgus angulation. At one year of follow-up, our patient had no pain and her elbow had a restored full range of motion. Furthermore, a radiographic examination revealed no signs of avascular necrosis, physeal arrest or angular deformity (Figures 9 and 10).

## Discussion

Hahn-Steinthal fracture of the capitellum is rarely seen in children younger than 12 years of age [3,5,7]. It is important to emphasize that these fractures can be misdiagnosed simply because of bad radiologic examinations, inexperience in the interpretation of capitellar fragments and the bizarre appearance of the fracture, especially if there is considerable rotation associated with the displacement. They can, however, lead to significant disability by limiting elbow motion if undiagnosed and untreated [4,5].

There are few references in the orthopedic literature to surgical treatment of these fractures in the pediatric population, and even less about internal fixation with cannulated screws [5-8,13]. Closed reduction has been particularly advocated in this population, but this can be difficult to achieve [2]. Internal fixation with Kirschner wire has been the historically preferable method of fixation, as the cartilaginous component of the fragment is often very large with a minimal amount of cancellous or subchondral bone [4,5,8]. However, Kirschner wires penetrate the articular surface, do not provide stable fixation and cast immobilization is mandatory for a long period. Cannulated screw fixation enables adequate interfragmentary reduction and compression. Contrary to pin or wire techniques, this type of fixation does not require further hospital admissions and the rehabilitation program starts earlier and is uninterrupted [2,3]. Headless double-threaded screws (Herbert type) could also be used [2,5]. However, we feel that for thin fragments this type of screw can fail to confer satisfactory fixation due to its compression mechanism. Headed screws sunk into the cartilage were chosen in the presented case due to previous experience and the implants available to the authors. Presently, the preferred method of treatment for isolated Hahn-Steinthal Type 1 capitellum fracture in adults is open reduction and internal fixation and a wide variety of techniques are described, such as Kirschner wires, compression screws, Herbert screws and biodegradable pins [2,3,10,12].

In our reported case, a large capitellum fragment displaced upwards and forwards was visualized on an oblique radiograph of her elbow. The method of treatment chosen by the authors, which was internal fixation with two cannulated screws, allowed them to achieve precise reduction of the large capitellum fragment and accurate placement of the screws where desired to offer good compression and stability. The excellent fixation obtained allowed our patient to start early active motion after cast removal, which occurred three weeks after surgery. It is important to emphasize that, as with most serious elbow injuries, this joint may take many months to regain a full range of motion. At one year follow-up, our patient had full recovery, no pain and a radiographic examination showed no signs of avascular necrosis, physeal arrest or angular deformity. She was also able to fully participate in sports without symptoms.

## Conclusion

A displaced Hahn-Steinthal type 1 capitellum fracture such as described must be anatomically reduced to restore articular congruity and to minimize potential disablement. This goal is more often achieved by open reduction and internal fixation, and cannulated screw fixation has many advantages. Being subarticular, it provides suitable interfragmentary compression and avoids incongruence, allowing early joint motion. It does not need to be removed.

The authors recommend open reduction and internal fixation with cannulated screws to best achieve these goals in the pediatric age group.

The authors recognize limits of this case report, due to a short term follow-up and the absence of an index score to classify the clinical and radiological evolution.

## Consent

Written informed consent was obtained from the mother of the patient for publication of this case report (the patient's legal guardian) and any accompanying images. The written consent is available for review by the Editor-in-Chief of this journal.

## Competing interests

The authors declare that they have no competing interests.

## Authors' contributions

JAGP was the main author and performed the clinical assessment, follow-up and the bibliographic research. APMF performed the clinical assessment, the surgery and the follow-up. APTCF performed the surgery and the bibliographic research. CA performed the clinical assessment and the surgery, and was a major contributor in writing the manuscript. MCR was a major contributor in writing the manuscript. MP was a major contributor in writing the manuscript. BTJ performed the clinical assessment and the bibliographic research. FF performed the clinical assessment and the bibliographic research. All authors have read and approved the final manuscript.
